# Comparing surgical outcomes of da Vinci SP and da Vinci Xi for endometrial cancer surgical staging in a propensity score-matched study

**DOI:** 10.1038/s41598-023-37659-z

**Published:** 2023-07-20

**Authors:** Ki Eun Seon, Yong Jae Lee, Jung-Yun Lee, Eun Ji Nam, Sunghoon Kim, Young Tae Kim, Sang Wun Kim

**Affiliations:** Division of Gynecologic Oncology, Department of Obstetrics and Gynecology, Institute of Women’s Life Medical Science, Yonsei University College of Medicine, 50 Yonsei-ro Seodaemun-gu, Seoul, 03722 Korea

**Keywords:** Surgical oncology, Endometrial cancer

## Abstract

The number of studies comparing robotic systems in endometrial cancer staging is limited. This retrospective study analyzed the medical records of 42 consecutive endometrial cancer patients, who underwent robotic staging using the da Vinci SP (SP) system, and 126 propensity score-matched patients who underwent staging using the da Vinci Xi (Xi) system. Median console and total operation times were longer in the SP group than those in the Xi group (125 vs. 77 min, p < 0.001; 225 vs. 154.5 min, p < 0.001, respectively). Notably, the median console time of the first 10 cases using SP was 184 min; it subsequently decreased to 99.5 min in the fourth 10 cases. SP had lesser postoperative hemoglobin (Hb) change (0.6 ± 0.7 g/dL vs. 1.8 ± 0.9 g/dL in Xi, p < 0.001) and lower median pain score at 6 h after surgery (2 vs. 3 in Xi, p = 0.046). Moreover, median postoperative hospital stay was shorter in the SP group (2 days) than that in the Xi group (6 days) (p < 0.001). Although SP was correlated with lower postoperative Hb change, shorter postoperative hospital stay, and lower pain score than those in Xi, it required longer operation times. Further prospective randomized studies are needed to validate the benefits of SP compared to other robotic platforms.

## Introduction

Endometrial cancer is the sixth most common cancer in women worldwide. The cumulative lifetime incidence risk of women aged 0–74 years is 1.05%^1^. The incidence of endometrial cancer is increasing owing to the high prevalence of obesity and aging in the population^2,3^. The standard treatment for early endometrial cancer is surgical staging, including total hysterectomy with bilateral salpingooophorectomy, and nodal assessment. Notable advances in minimally invasive surgery (MIS) have enabled laparoscopic and robotic surgical staging in gynecological oncology. In endometrial cancer, MIS is preferred because of its lower postoperative morbidity rate, reduced hospital stay, and improved quality of life^4^. Since the United States Food and Drug Administration approved the da Vinci Surgical System (Intuitive Surgical, Sunnyvale, CA, USA) for gynecology in 2005, the use of robotic surgery in gynecological diseases has greatly increased^5^. In endometrial cancer, similar survival outcomes, shorter hospital stays, less estimated blood loss (EBL), and lower complication rate were observed with robot-assisted staging surgery than those with conventional laparoscopy and laparotomy^6–8^. In the recently introduced da Vinci SP system (SP), the two-joint articulation of instruments enables more powerful manipulation while reducing collision between instruments, compared to that by single-site robotic systems,^9^. In addition, the flexible camera provides a new operating angle in all directions. However, in the field of gynecological oncology, no study till date has compared surgical outcomes between the SP and da Vinci Xi system (Xi). This study aimed to evaluate the safety and feasibility of SP in terms of intra- and postoperative complications and compare the perioperative surgical outcomes between SP and Xi in the surgical staging of endometrial cancer.

## Results

### Demographic characteristics of patients

**Figure 1:**
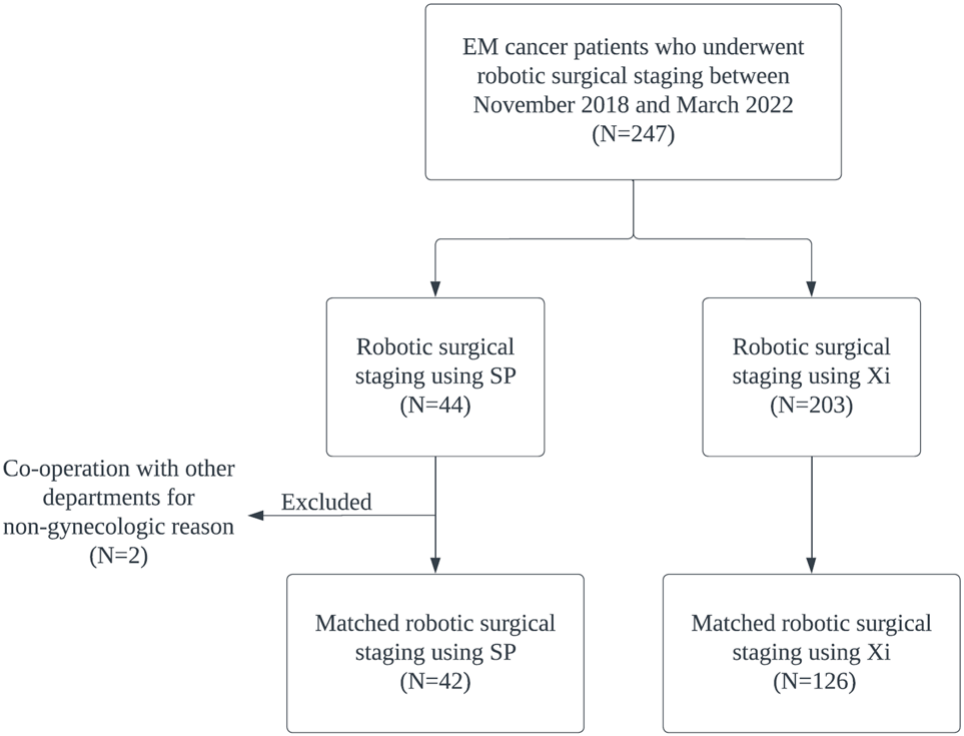
Flowchart of selected endometrial cancer patients, who underwent robotic surgical staging between November, 2018 and March, 2022, using the da Vinci SP (SP) and da Vinci Xi systems (Xi).

Between November, 2018 and March, 2022, 247 patients with endometrial cancer underwent robotic surgical staging using SP and Xi. Two patients were excluded because they underwent surgeries with other surgical departments for nongynecologic reasons (breast mass excision and total thyroidectomy). Of the 245 patients, 42 (17.1%) underwent robotic surgical staging using SP and 203 (82.9%) using Xi. The propensity-score matching (PSM) technique with four covariates (age, International Federation of Gynecology and Obstetrics [FIGO] stage 2009, histologic type, and grade) and a 1:3 ratio (SP group: Xi group) were used. These four covariates used in this study are composed of important prognostic factors in endometrial cancer^10^. A total of 42 patients in the SP group were matched with 126 patients in the Xi group (Fig. 1). The baseline patient characteristics, before and after PSM, were described using summary statistics. Patients in the SP and Xi groups were well balanced in terms of matched covariates (Supplementary table 1).

The baseline characteristics of all included patients are shown in Table 1. The patients’ demographic characteristics, including age, body mass index (BMI), gravidity, parity, menopausal status, previous abdominal surgery (i.e., cesarean section, myomectomy, and appendectomy), and American Society of Anesthesiologists (ASA) class (assessment of patient’s preanesthesia medical comorbidities) were similar in both groups.

**Table 1:** Demographic and clinicopathological characteristics of endometrial cancer patients who underwent robotic surgical staging using da Vinci SP (n = 42) or Xi system (n = 126).

	SP (n = 42)	Xi (n = 126)	*p-value*
Age, years, Mean ± SD	48.7 ± 8.8	48.6 ± 8.7	0.927
Body mass index, kg/m^2^, Mean ± SD	24.4 ± 4.7	25.3 ± 5.1	0.274
Gravidity, Median (IQR)	2 (1–3)	2 (1–3)	0.734
Parity, Median (IQR)	2 (0–2)	2 (0–2)	0.683
Menopause, n (%)	18 (42.8)	41 (32.5)	0.225
Previous abdominal surgery, n (%)	14 (33.3)	34 (27.0)	0.430
ASA class, n (%)			0.979
I	7 (16.7)	22 (17.5)	
II	31 (73.8)	91 (72.2)	
III	4(9.5)	13 (10.3)	
FIGO stage, n (%)			0.708
I	38 (90.4)	110 (87.3)	
II	2 (4.8)	4 (3.2)	
III	2 (4.8)	11 (8.7)	
IV	0	1 (0.8)	
Histologic type, n (%)			
Endometrioid adenocarcinoma	42 (100)	126 (100)	
Others	0	0	
Grade, n (%)			0.576
G1	25 (59.5)	67 (53.2)	
G2	13 (31.0)	50 (39.7)	
G3	4 (9.5)	9 (7.1)	
Myometrial invasion, n (%)			0.611
< 1/2	37 (88.1)	107 (84.9)	
≥ 1/2	5 (11.9)	19 (15.1)	
Lymphovascular space invasion, n (%)	1 (2.4)	20 (15.9)	0.022
Uterus weight, g, Mean ± SD	157.1 ± 68.3	149.8 ± 111.8	0.691
Abdominal adhesion, n (%)	21 (50.0)	38 (30.2)	0.020
Postoperative treatment			
CTx, n (%)	4 (9.5)	7 (5.6)	
T + C	3 (7.1)	6 (4.8)	
Other regimen	1 (2.4)	1 (0.8)	
Total cycle, median (range)	5 (4–6)	6 (3–6)	
RTx, n (%)	3 (7.1)	24 (19.0)	
Vaginal	2 (4.8)	22 (17.5)	
Whole pelvis + Vaginal	1 (2.4)	2 (1.6)	
CTx + RTx, n (%)	1 (2.4)	7 (5.6)	

*ASA,* American Society of Anesthesiologists; *SD,* standard deviation; *IQR,* interquartile range; *FIGO,* International Federation of Gynecology and Obstetrics; *CTx,* chemotherapy; *RTx,* radiation therapy; *T* + *C,* Paclitaxel with Carboplatin.

### Clinicopathological characteristics

Clinicopathological characteristics of the patients are presented in Table 1. There were no significant differences in FIGO stage, histologic type, grade, myometrial invasion, or uterus weight. Most patients in both groups had FIGO stage I and grade 1. Through PSM, all included patients were found to be of the endometrioid histologic type. The lymphovascular space invasion (LVSI) rate was higher in the Xi group (2.4% and 15.9% for SP and Xi, respectively, p = 0.022). The incidence of abdominal adhesions was higher in the SP group (50.0% and 30.2% for SP and Xi, respectively, p = 0.020).

### Perioperative surgical outcomes

**Figure 2:**
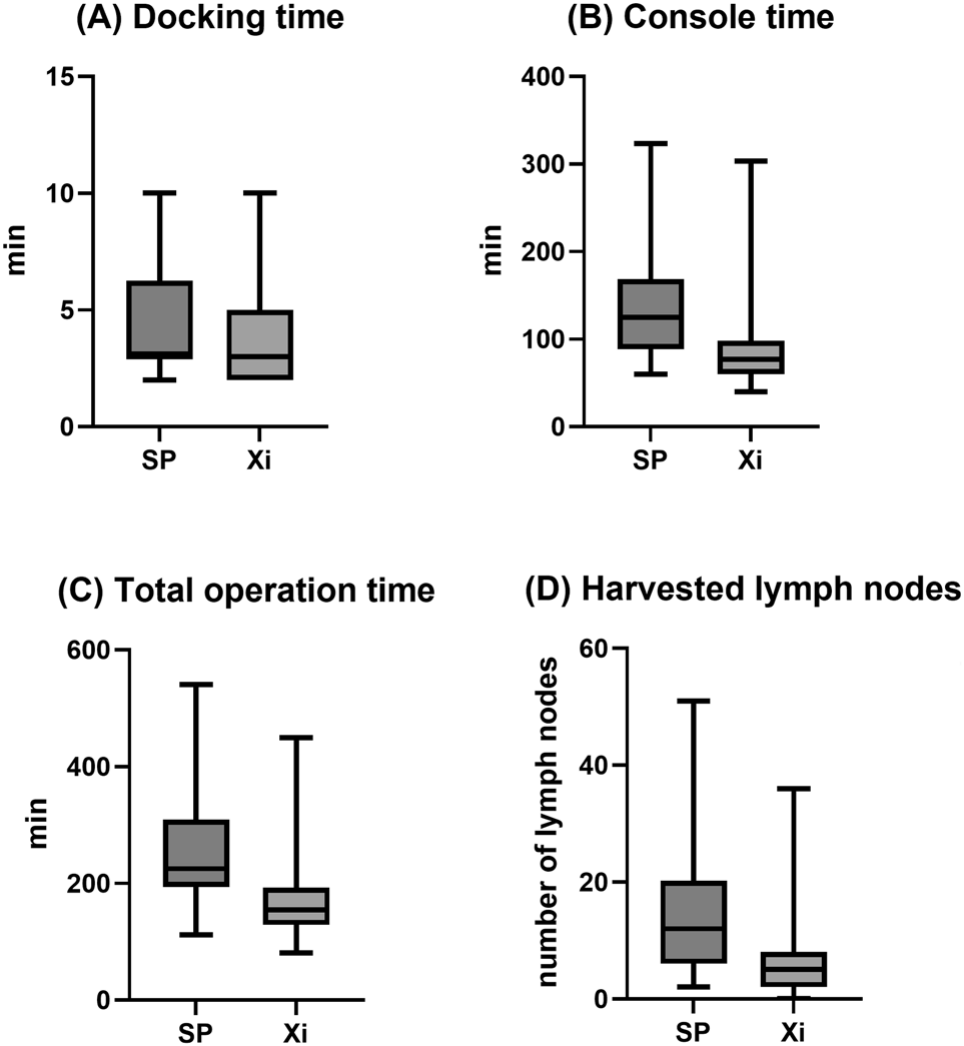
(**A**) Docking time, (**B**) console time, (**C**) total operation time and (**D**) harvested lymph nodes of endometrial cancer patients, who underwent robot surgical staging using the da Vinci SP (SP) and da Vinci Xi system (Xi) systems (min to max, interquartile range).

There was no significant difference in median (range) docking time and EBL between the two groups (3 [2–10] vs. 3 [2–10] min for SP and Xi, respectively, p = 0.247; 30 [10–200] vs. 30 [10–300] mL for SP and Xi, respectively, p = 0.575) (Table 2). However, median (range) console and total operation times were significantly longer in the SP group (125 [60–323] vs. 77 [44–303] min for SP and Xi, respectively, p =  < 0.001; 225 [112–540] vs. 154.5 [81–449] min for SP and Xi, respectively, p =  < 0.001) (Fig. 2A–C). In the subgroup analysis, comparing the 31st to 40th patient in the SP group (subgroup 4) with the Xi group, the difference in mean docking, console, and total operation times was reduced (Supplementary Fig. 1). Intraoperative and postoperative complications did not differ between the two groups (4.8 vs. 1.6% for SP and Xi, respectively, p = 0.260; 14.3 vs. 13.5% for SP and Xi, respectively, p = 0.897, respectively). Vaginal wall laceration and bowel injuries (duodenal and rectal serosa laceration) were included in intraoperative complications. Postoperative complications included respiratory complication, fever requiring admission, ileus, lymphedema, lymphocele, lymphorrhea, hydronephrosis, and others (vaginal vault bleeding, brachial plexopathy, abdominal pain, acute postoperative parotitis, benign paroxysmal positional vertigo, and acute vestibular neuritis). Seven patients in the Xi group required postoperative transfusion. There was no significant difference in postoperative transfusion and mean preoperative hemoglobin (Hb) between the SP and Xi groups (0 vs. 5.6% for SP and Xi, respectively, p = 0.194; 12.0 ± 1.6 vs. 12.6 ± 1.7 g/dL for SP and Xi, respectively, p = 0.056). Mean postoperative Hb was high (mean ± standard deviation, 11.4 ± 1.4 vs. 10.8 ± 1.4 g/dL for SP and Xi, respectively, p = 0.021), while the postoperative Hb change (defined as the difference between preoperative and postoperative day 1 Hb) was less (0.6 ± 0.7 vs. 1.8 ± 0.9 g/dL for SP and Xi, respectively, p =  < 0.001) in the SP group. In addition, the median (interquartile range, IQR) postoperative hospital stay was shorter in the SP group (2 [IQR: 2.0–3.0] vs. 6 [IQR: 3.0–7.0] days for SP and Xi, respectively, p =  < 0.001). No case required additional port or conversion to other mode of surgery in either group.

**Table 2:** Propensity score-matched comparison of perioperative surgical outcomes between the da Vinci SP (n = 42) and Xi (n = 126) surgical systems.

Perioperative surgical outcomes	SP (n = 42)	Xi (n = 126)	*p-value*
Docking time^a^, min, median (range)	3 (2–10)	3 (2–10)	0.247
Console time^b^, min, median (range)	125 (60–323)	77 (44–303)	< 0.001
Total operation time^c^, min, median (range)	225 (112–540)	154.5 (81–449)	< 0.001
Estimated blood loss, mL, median (range)	30 (10–200)	30 (10–300)	0.575
Preoperative Hb, g/dL, Mean ± SD	12.0 ± 1.6	12.6 ± 1.7	0.056
Postoperative Hb, g/dL, Mean ± SD	11.4 ± 1.4	10.8 ± 1.4	0.021
Postoperative Hb change^d^, g/dL, mean ± SD	0.6 ± 0.7	1.8 ± 0.9	< 0.001
Transfusion^e^, n (%)	0	7 (5.6)	0.194
Intraoperative complications, n (%)	2 (4.8)	2 (1.6)	0.260
Bowel injury	2 (4.8)	1 (0.8)	
Vaginal wall laceration	0	1(0.8)	
Postoperative complications^f^, n (%)	6 (14.3)	17 (13.5)	0.897
Respiratory complication	–	1(0.8)	
Fever requiring admission	2 (4.8)	2 (1.6)	
Ileus	0	2 (1.6)	
Wound complication (incisional hernia)	0	0	
Lymphedema/Lymphocele/Lymphorrhea	1 (2.4)	6 (4.8)	
Hydronephrosis	0	2 (1.6)	
Others (e.g. vaginal vault bleeding, brachial plexopathy)	3 (7.1)	4 (3.2)	
Conversion to other mode of surgery, n (%)	0	0	
Postoperative hospital stay, days, median (IQR)	2 (2–3)	6 (3–7)	< 0.001

*SD,* standard deviation; *Hb,* hemoglobin; *IQR,* interquartile range.

^a^Docking time is defined as the time to move the robotic cart towards the surgical field and attach robotic arms to the inserted ports.

^b^Console time is defined as the time taken for the surgeon to perform the operation at the console.

^c^Total operation time is defined as the time from the initial skin incision for port insertion to the closure of skin incision.

^d^Postoperative Hb change is defined as Hb difference between preoperative and postoperative day 1.

^e^All transfusions were postoperative cases. ^f^ Postoperative complications were evaluated during the first month after surgery.

### Perioperative surgical outcomes in the SP group according to a continuous chronological order

Since SP was more recently introduced, it was necessary to analyze the change in surgical outcomes over time in consideration of the learning curve-related effect. The perioperative surgical outcomes of SP were divided into four subgroups, with 10 patients each in succession, according to a continuous chronological order (from the 1st to 40th patient with endometrial cancer who underwent robotic surgical staging using SP).

**Figure 3:**
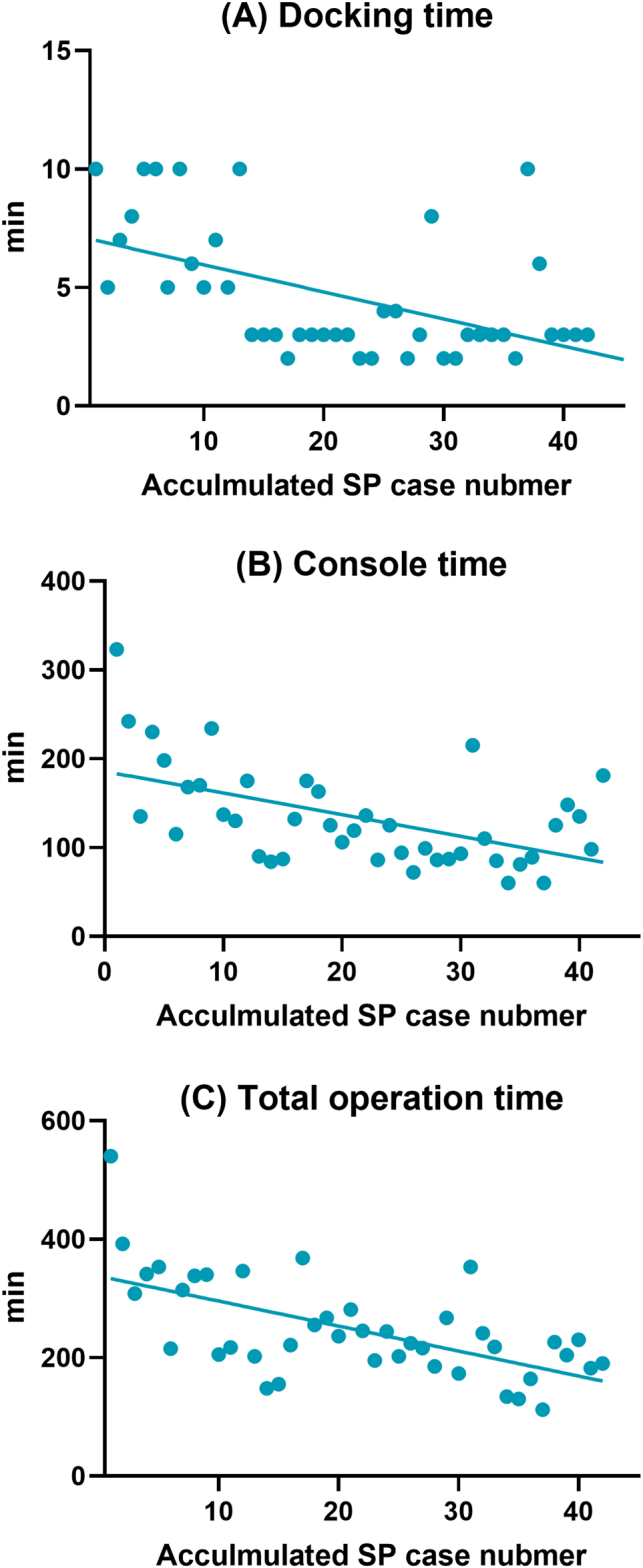
Perioperative surgical outcomes in the da Vinci SP (SP) group arranged in a chronological order.

The perioperative surgical outcomes of each subgroup are presented in Table 3. Docking, console, and total operation times generally tended to decrease with increasing surgical experience (Fig. 3). The median docking and console times decreased gradually until subgroup 3 (7.5 and 184 min in the subgroup 1, 3 and 127.5 min in the subgroup 2, 3 and 93.5 min in the subgroup 3, and 3 and 99.5 min in the subgroup 4). During the entire study period, median total operation time decreased (339 min in the subgroup 1, 228.5 min in the subgroup 2, 220 min in the subgroup 3, and 211 min in the subgroup 4). The median (range) docking, console, and total operation times decreased significantly in subgroup 4 compared with those of subgroup 1 (7.5 [5–10] vs. 3 [2–10] min for subgroup 1 and 4, respectively, p = 0.003; 184 [115–323] vs. 99.5 [60–215] min for subgroup 1 and 4, respectively, p = 0.004; 339 [205–540] vs. 211 [112–353] min for subgroup 1 and 4, respectively, p = 0.009, respectively). However, no significant tendency was observed for median (range) EBL (40 [10–200] vs. 50 [20–100] mL for subgroup 1 and 4, respectively, p = 0.590), mean postoperative Hb level change (0.8 ± 0.7 vs. 0.4 ± 0.7 g/dL for subgroup 1 and 4, respectively, p = 0.120), and median hospital stay (3 [IQR: 2–4] vs. 2 [IQR: 1–2] days for subgroup 1 and 4, respectively, p = 0.070) between subgroups 1 and 4.

**Table 3:** Perioperative surgical outcomes of patients who underwent surgical staging using the da Vinci SP surgical system.

Perioperative surgical outcomes	SP subgroup 1 (n = 10)	SP subgroup 2 (n = 10)	SP subgroup 3 (n = 10)	SP subgroup 4 (n = 10)
Docking time^a^, min, median (range)	3 (2–10)	7.5 (5–10)	3 (2–10)	3 (2–8)
Console time^b^, min, median (range)	125 (60–323)	184 (115–323)	127.5 (84–175)	93.5 (72–136)
Total operation time^c^, min, median (range)	225 (112–540)	339 (205–540)	228.5 (148–368)	220 (173–281)
Estimated blood loss, mL, median (range)	30 (10–200)	40 (10–200)	30 (10–60)	30 (20–50)
Postoperative Hb change^d^, g/dL, mean ± SD	0.64 ± 0.69	0.80 ± 0.65	0.85 ± 0.98	0.47 ± 0.37
Transfusion, n (%)	0	0	0	0
Intraoperative complications, n	1	0	1	0
Bowel injury	1	0	1	0
Other	0	0	0	0
Postoperative complications^e^, n	4	1	0	1
Respiratory complication	0	0	0	0
Fever requiring admission	1	0	0	1
Lymphedema/Lymphocele/Lymphorrhea	1	0	0	0
Hydronephrosis	0	0	0	0
Other	2	1	0	0
Conversion to other mode of surgery, n	0	0	0	0
Postoperative hospital stay, days, median (IQR)	3 (2–4)	2 (1.75–3)	2 (2–2.25)	2 (1–2)

The patients were divided into four subgroups, with 10 patients each in succession, arranged in a chronological order.

*SD,* standard deviation; *Hb,* hemoglobin; *IQR,* interquartile range.

^a^Docking time is defined as the time to move the robotic cart towards the surgical field and attach robotic arms to the inserted ports.

^b^Console time is defined as the time taken for the surgeon to perform the operation at the console.

^c^Total operation time is defined as the time from the initial skin incision for port insertion to the closure of skin incision.

^d^Postoperative Hb change is defined as Hb difference between preoperative and postoperative day 1.

^e^Postoperative complications were evaluated during the first month after surgery.

#### Frailty index assessment related to perioperative complications

Additionally, we conducted an analysis of the age-adjusted Charlson comorbidity index score (A-CCI) within the study population, considering the significance of frailty assessment in evaluating perioperative complications in the field of gynecologic oncology^11,12^. Among the total number of patients included in the study, four patients in the SP group and five patients in the Xi group had A-CCI scores ≥ 3. However, no significant difference was observed in the selection of robotic surgical systems between SP and Xi based on A-CCI (p = 0.109). In the SP group, the intra-/postoperative complication rate was 18.4% for patients with A-CCI < 3 and 25% for patients with A-CCI ≥ 3. In the Xi group, the intra-/postoperative complication rate was 11.6% for patients with A-CCI < 3 and 40% for patients with A-CCI ≥ 3. Nonetheless, when analyzing the entire patient cohort, no significant correlation was found between A-CCI and the intra-/postoperative complication rate (p = 0.089).

### Characteristics of lymph node resection

The two-step indocyanine green (ICG) sentinel lymph node (SLN) mapping method was performed in the SP group (42.9%), while most of the patients in Xi group underwent the one-step method (73.8%) (Table 4). The proportion of pelvic lymph node (LN) dissection (LND), with paraaortic LND, was higher in the SP group (85.7 vs. 72.2% for SP and Xi, respectively, p =  < 0.001). The median (range) number of harvested LNs was 12 (2–51) in the SP group and 5 (0–36) in the Xi group (Fig. 2-D). There was no significant difference in LN metastasis between the two groups.

**Table 4:** Pelvic and paraaortic lymph node clinicopathological characteristics of the da Vinci SP and Xi endometrial cancer surgical staging.

	SP (n = 42)	Xi (n = 126)	*p-value*
ICG sentinel LN mapping, n (%)			< 0.001
No sentinel LN mapping	15 (35.7)	13 (10.3)	
One-step	9 (21.4)	93 (73.8)	
Two-step	18 (42.9)	20 (15.9)	
Range of LND, n (%)			< 0.001
No LND	–	8 (6.4)	
PLND only	6 (14.3)	27 (21.4)	
PLND with PALND	36 (85.7)	91 (72.2)	
Number of harvested LNs, Median (range)			
Total	12 (2–51)	5 (0–36)	< 0.001
PLN	6 (2–36)	4 (0–30)	
PALN	5 (0–27)	0 (0–22)	
LN metastasis, n (%)			0.139
No metastasis	40 (95.2)	107 (84.9)	
LN metastasis	2 (4.8)	8 (6.4)	
Only PLN	1 (2.4)	6 (4.8)	
Only PALN	0 (0)	1 (0.8)	
PLN and PALN	1(2.4)	1 (0.8)	
Not available nodal status	0 (0)	11 (8.7)	

*LN,* lymph node; *LND,* lymph node dissection; *PLND,* pelvic lymph node dissection; *PALND,* para-aortic lymph node dissection; *PLN*, pelvic lymph node; *PALN*, paraaortic lymph node; *ICG,* indocyanine green.

### Postoperative pain

The patient-controlled analgesia (PCA) administration rate was significantly lower in the SP group (9.5% in SP vs. 43.7% in Xi, respectively, p =  < 0.001) (Table 5). Although intravenous (IV) PCA was administered in a high proportion of patients in the Xi group, the median (IQR) postoperative pain score at 6 h after surgery was significantly lower in the SP group (2 [0–4] in SP vs. 3 [0–8] in Xi, respectively, p = 0.046). However, the pain scores at 12 h and 24 h after surgery were not significantly different between the two groups. Additionally, the IV analgesic requirement rate (57.1% in SP vs. 44.4% in Xi, respectively, p = 0.154) and median (range) number of additional IV analgesics (2 [0–7] in SP vs. 2 [0–8] in Xi, respectively, p = 0.822) were not different between the two groups.

**Table 5:** Pain score during postoperative 24 h, and usage of pain controller in patient who underwent robot surgical staging using the da Vinci SP and Xi surgical systems.

	SP (n = 42)	Xi (n = 126)	*p-value*
Pain score^a^, median(range)			
At 6 h	2 (0–4)	3 (0–8)	0.046
At 12 h	2 (0–5)	2 (0–7)	0.455
At 24 h	2 (0–4)	2 (0–6)	0.372
Patients with IV PCA, n (%)	4 (9.5)	55 (43.7)	< 0.001
Additional IV analgesics requirement, n (%)	24 (57.1)	56 (44.4)	0.154
Number of additional IV analgesics requirement, median (range)^a^	2 (0–7)	2 (0–8)	0.822

*PCA,* Patient-controlled analgesia; *IV*, intravenous.

^a^Numeral rating scale.

### Postoperative treatment, recurrence rate, and survival outcomes

The median follow-up (defined from the date of surgery to the latest out-patient visit) duration for the SP and Xi groups was 13 months (range 1–44 months) and 22 months (range 4–42 months), respectively. During the postoperative treatment, four and seven patients received adjuvant chemotherapy in the SP and Xi groups, respectively (Table 1). Three patients (7.1% of SP patients) in the SP group and approximately 1.7 times more percentage of patients in the Xi group (24 patients, 19.0% of Xi patients) received postoperative radiation therapy. Among both the groups, only two patients in the Xi group developed recurrence; however, no death was reported.

## Discussion

This study compared the perioperative surgical outcomes of two robotic systems (SP and Xi) in surgical staging of endometrial cancer. To the best of our knowledge, this is the first study to compare the surgical outcomes of SP and Xi in gynecologic oncology.

The mean total operation time of a recent multicenter retrospective study that included da Vinci robotic systems (150 min, n = 598) was similar to that of Xi^13^. Moreover, intraoperative and postoperative complication rates in our study were not different from the multicenter retrospective study (5.02 and 14.38%, respectively). In another single-institution retrospective study comparing robotic surgical staging of da Vinci Si and Xi in early-stage endometrial cancer, the total operative time (181.9 min) and intraoperative complication rate (1.8%) in Xi were similar to those in our study^14^. Because SP has been introduced recently, no study has compared the surgical outcomes of SP with those of other robotic systems for endometrial cancer.

In this study, the median console and total operation times were significantly longer in SP than those in Xi. Several plausible hypotheses can explain these differences.

1. Until October, 2018, da Vinci Si and Xi were used for all robotic surgeries at our institution. Since the introduction of SP in November, 2018, we have performed robotic surgery for benign or malignant gynecological tumors using the new system. This study included initial cases of robotic endometrial cancer staging using SP. Similar to the initial surgical outcomes of other robotic systems, those of SP were also affected by the learning curve^15–17^. These tendencies were well described in our subgroup analysis, which divided the SP group into four subgroups.
2. The feasibility and accurate predictive value of SLN mapping in early endometrial cancer were observed in systemic reviews and randomized clinical trials^18–20^. The firefly fluorescence imaging system, which was not available in the SP during our study period, enabled SLN mapping in Xi. To overcome this limitation, our institution has introduced an assisting imaging system with an endoscopic near-infrared fluorescence camera (PINPOINT, Novadaq Technologies Inc., Toronto, Ontario, Canada) using the TilePro function of the da Vinci SP system (Supplementary Fig. 2). In addition, the two-step method was performed in SP (42.9%), with higher median number of harvested LNs than that of Xi (12 vs. 5 for SP and Xi, respectively, p < 0.001). LND can vary depending on the patient’s clinical risk and surgeon’s discretion, thereby increasing the operation time. These additional preparations and different SLN mapping methods also seemed to have affected the increase in operation time for SP.
3. The fascia was generally not repaired in Xi because an 8 mm trocar was used. However, as the SP has an incision of approximately 2.5 cm at the umbilicus, fascia layer approximation should be performed to prevent incisional hernia^21^. The incision site was closed with interrupted sutures (Vicryl™ 1-0, Ethicon Endo-surgery, Cincinnati, Ohio, USA). This could also increase the operation time in SP.

Lower mean postoperative Hb level change, shorter postoperative hospital stays, and lower pain score 6 h after surgery were observed in the SP group than those in the Xi group. Other studies comparing single-site and multiport robotic systems were reviewed to determine whether these differences are related to the number of ports. In a retrospective study that compared single-site and multiport robotic surgeries in endometrial cancer patients, there were no significant differences between the two groups^22^. In another case–control study, lower EBL and shorter hospital stay were observed in robotic single-site hysterectomy than those in multiport hysterectomy for early endometrial cancer^23^. However, as the number of patients was relatively small, the EBL and hospital stays did not appear to be clinically significant.

In this study, seven patients in Xi group received transfusion. All the transfusions were during postoperative periods. Although the median and range of EBL were not significantly different between the two groups, postoperative Hb change was significantly lower in the SP group than that in the Xi group. Since the postoperative Hb change was defined as Hb difference between preoperative and postoperative day 1, the degree of Hb change and proportion of transfusion may not exactly match. For example, three patients received transfusion on the immediate postoperative day, and one patient received on postoperative day 3. Nevertheless, the postoperative Hb change was a reliable measurement showing significant difference between the two groups.

Although IV PCA administration rate was significantly lower in the SP group, the median postoperative 6-h numerical rating scale (NRS) was lower in the SP group than that in the Xi group. Not only after 6 h, but the overall range of pain was also less in the SP group. This difference was assumed to be affected by the number of ports and sites (risk of abdominal muscle damage). Moreover, periumbilical infiltration of bupivacaine during repair of the umbilical incision of SP could affect the decrease in postoperative pain score in SP^24,25^.

Postoperative hospital stay was shorter in the SP group than that in the Xi group (2 vs. 6 days for SP and Xi, respectively, p < 0.001). Although the decision on the patient’s discharge was made entirely at the surgeon’s discretion, the general discharge criteria included tolerable postoperative pain (i.e., NRS < 4), no postoperative fever for more than 24 h, and no other clinical condition requiring additional medical treatment^26^. The postoperative pain, transfusion rate, and perioperative complications could be related to the postoperative hospital stay.

Moreover, it is important to acknowledge that not all patients in this study underwent the essential immunohistochemistry staining and polymerase chain reaction testing for molecular classification. Furthermore, given the retrospective design of our study, which primarily emphasized perioperative surgical outcomes, conducting a thorough and detailed analysis was not within the scope of our investigation. Nevertheless, it is worth highlighting the potential significance of future research endeavors involving additional molecular classification tests and their subsequent analysis. Such efforts would be particularly valuable in the context of tailoring adjuvant treatment approaches based on the individual molecular and genomic profiling of patients within the study cohort^27^.

The strength of our study was that it was the first to compare robotic endometrial cancer staging using SP and Xi. Since SP’s introduction, limited studies have compared the two robotic systems in the field of gynecology. The present study is a single-institution retrospective study with a small number of patients and was also limited by a relatively short follow-up period. In addition, although most surgeons were trained at the same institution, bias resulting from the variability of each surgeon could not be completely excluded. In conclusion, despite these limitations, our study suggests that robotic endometrial cancer surgical staging using SP appears to be safe and feasible in terms of intra- and postoperative complications. Although SP had a longer console and total operation time than Xi, these values tended to decrease significantly during the learning curve as surgical experience increased. In addition, SP system showed less postoperative Hb change, pain at 6 h after surgery, and hospital stay compared to those with the Xi system. Prospective randomized studies are required to verify the advantages of SP over other robotic platforms.

## Materials and methods

### Patient

We retrospectively reviewed 247 consecutive patients with endometrial cancer, who underwent robotic surgical staging using SP and Xi between November, 2018 and March, 2022, at Women’s Cancer Center, Yonsei Cancer Center, Seoul, Korea (Fig. 1). Through previous pathological confirmation, all included patients were diagnosed with endometrial cancer before planning the robotic surgical staging.

### Preoperative and intraoperative measures

Preoperative measurements included age, BMI, gravity, parity, menopausal status, comorbidities, history of prior abdominal surgery, ASA’s class of physical status, preoperative Hb level, histologic type, and grade. Intraoperative measures collected during the surgical procedures included robot docking time (defined as the time to move the robotic cart towards the surgical field and attach robotic arms to the inserted ports), console time (defined as the time taken for the surgeon to perform the operation at the console), total operation time (defined as the time from the initial skin incision for port insertion to the closure of skin incision), abdominal adhesion, conventional LN dissection or SLN biopsy, intraoperative complications, intraoperative transfusion rate, EBL during the operation, and conversion to other modes of surgery.

### Surgical procedures

When clinically necessary, the patients underwent complete surgical staging, including total hysterectomy, bilateral salpingooophorectomy, conventional LN dissection or SLN biopsy (pelvic with/without paraaortic LN), and other procedures. All surgical staging procedures using SP and Xi were performed by six experienced gynecologic oncology surgeons, familiar with MIS and robotic surgery, at a single tertiary referral hospital. Robotic system selection mainly depended on the surgeon’s discretion, and availability of the robotic platform at the scheduled operating date. Details of each robotic surgical procedure have been previously described by our group^21,28^.

#### LN resection

The selection of the LN resection method (conventional LN dissection or SLN biopsy, pelvic with or without paraaortic LN) was on the basis of risk categories, determined by preoperative histologic type and preoperative clinical stage from the magnetic resonance imaging scans and the surgeon’s consideration. SLN mapping using fluorescent imaging with ICG was performed through either a one- (cervical injection) or two-step (bilateral uterine cornual area and cervical injection) process^29^.

### Postoperative measures and follow-up

Postoperative data collected during the study period included postoperative day 1 Hb level, postoperative transfusion rate, postoperative pain assessment, the number of IV PCA administered, additional IV analgesic requirement until discharge, postoperative hospital stay (defined as the number of days from operation to discharge), postoperative complications during the 1st month after surgery, pathology reports (e.g., uterus weight, histologic type, grade, myometrial invasion, LVSI, and LN metastasis), postoperative treatment with overall response, disease recurrence, and death until September, 2022.

#### 6-, 12- and 24-h postoperative pain assessment

The Yonsei Cancer Center has adopted the Enhanced Recovery After Surgery guidelines since 2019^30^; accordingly, multimodal perioperative care has been applied to control pain, including nonsteroidal antiinflammatory drugs (NSAIDs), acetaminophen, gabapentin, and dexamethasone. All patients included in this study were routinely administered IV NSAIDs three times a day for 24 h after surgery; after 24 h they were changed to oral analgesics. In addition, IV PCA and analgesics (tramadol and pethidine) were administered according to the patient’s needs. Postoperative pain assessment, performed in this study, was evaluated on a NRS of 0–10 and reported 6, 12, and 24 h after the surgery.

#### One-week postoperative assessment and follow-up

Approximately 1 week after discharge, all patients visited the out-patient clinic for a regular postoperative assessment. In some patients, adjuvant chemotherapy and/or radiation were planned according to the final pathologic results. For other patients, regular follow-up was planned every 3 months for surveillance.

### Statistical analyses and PSM

Statistical analyses were performed using the Statistical Package for the Social Sciences Statistics for Windows (version 26.0; SPSS Inc., Chicago, Ill., USA). Continuous variables were compared using student’s t-test and the Mann–Whitney test. The Pearson’s chi-squared and Fischer’s exact tests were used to compare categorical variables. A two-sided p-value < 0.05 was considered to be statistically significant.

PSM was used to correct for potential confounding by bias. Propensity scores were estimated using logistic regression with four covariates, including age, FIGO stage, histologic type, and grade. Matching was performed according to the nearest matching pattern and a 1:3 ratio (SP group:Xi group). Detailed baseline patient characteristics, before and after matching, were described using summary statistics.

### Ethical approval

The Institutional Review Board (IRB) of Yonsei University approved this single-center retrospective study (IRB No. 4-2022-0665; July 13, 2022), conducted in compliance with the relevant guidelines and regulations of the IRB. Anonymous patient data was collected, and informed consent for this retrospective study was waived in accordance with the IRB policy of Yonsei University.

## Supplementary Information

Supplementary Information.

## Data Availability

Due to privacy and ethical concerns, neither the data nor the source of the data can be made available. Correspondence and requests for materials should be addressed to S.W.K.
