# Supplementary material for: Comparing surgical outcomes of da Vinci SP and da Vinci Xi for endometrial cancer surgical staging in a propensity score-matched study
Source: Sci Rep. 2023 Jul 20;13:11752. doi: 10.1038/s41598-023-37659-z (PMC10359395; doi:10.1038/s41598-023-37659-z)
Supplement: Supplementary file 1 — Supplementary Information. [file 41598_2023_37659_MOESM1_ESM.docx]

**Supplementary table 1.**

Baseline patient characteristics before and after propensity-score matching of endometrial cancer patients, who underwent robotic surgical staging using the da Vinci SP (SP) or da Vinci Xi systems (Xi)

| Characteristics | Before matching | | After matching | |
| --- | --- | --- | --- | --- |
|  | SP (n = 42) | Xi (n = 203) | SP (n = 42) | Xi (n = 126) |
| Age, years, Mean ± SD | 48.7 ± 8.8 | 52.2 ± 9.3 | 48.7 ± 8.8 | 48.6 ± 8.7 |
| FIGO stage, n (%) |  |  |  |  |
| Ⅰ | 38 (90.4) | 173 (85.2) | 38 (90.4) | 110 (87.3) |
| Ⅱ | 2 (4.8) | 9 (4.4) | 2 (4.8) | 4 (3.2) |
| Ⅲ | 2 (4.8) | 19 (9.4) | 2 (4.8) | 11 (8.7) |
| Ⅳ | - | 2 (1) | - | 1 (0.8) |
| Histologic type, n (%) |  |  |  |  |
| Endometrioid adenocarcinoma | 42 (100) | 190 (93.6) | 42 (100) | 126 (100) |
| Serous | - | 3 (1.5) | - | - |
| Clear | - | 3 (1.5) | - | - |
| Endometrial stromal sarcoma (ESS) | - | 5 (2.4) | - | - |
| MMT (carcinosarcoma) | - | 1 (0.5) | - | - |
| Mucinous | - | 1 (0.5) | - | - |
| Grade, n (%) |  |  |  |  |
| G1 | 25 (59.5) | 80 (39.4) | 25 (59.5) | 67 (53.2) |
| G2 | 13 (31.0) | 92 (45.3) | 13 (31.0) | 50 (39.7) |
| G3, clear | 4 (9.5) | 31 (15.3) | 4 (9.5) | 9 (7.1) |

*FIGO,* International Federation of Gynecology and Obstetrics

Each group were matched based on age, stage, histologic type, and grade.

**Supplementary Figures**


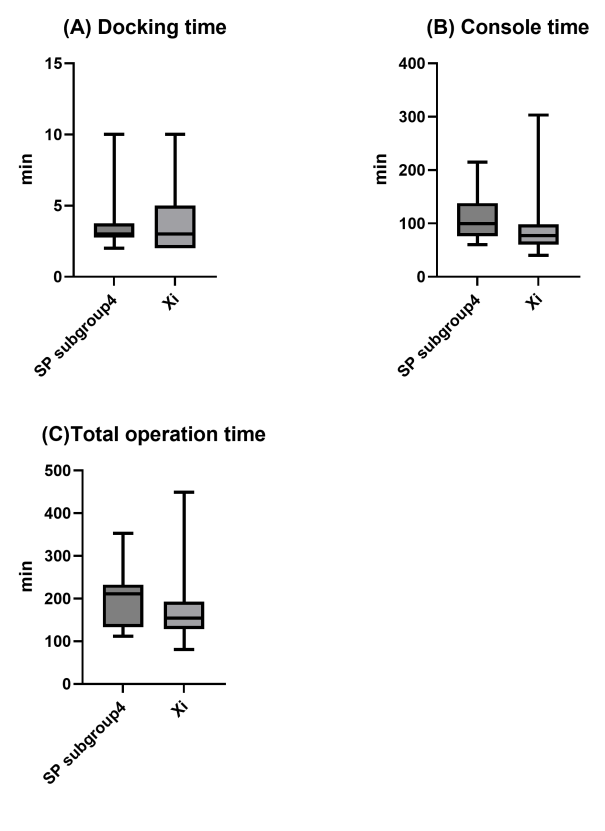


**Supplementary figure 1.**

Perioperative surgical outcomes in the SP subgroup 4 and the Xi group (min to max, IQR).

*SP*, da Vinci SP system; *IQR*, interquartile range.


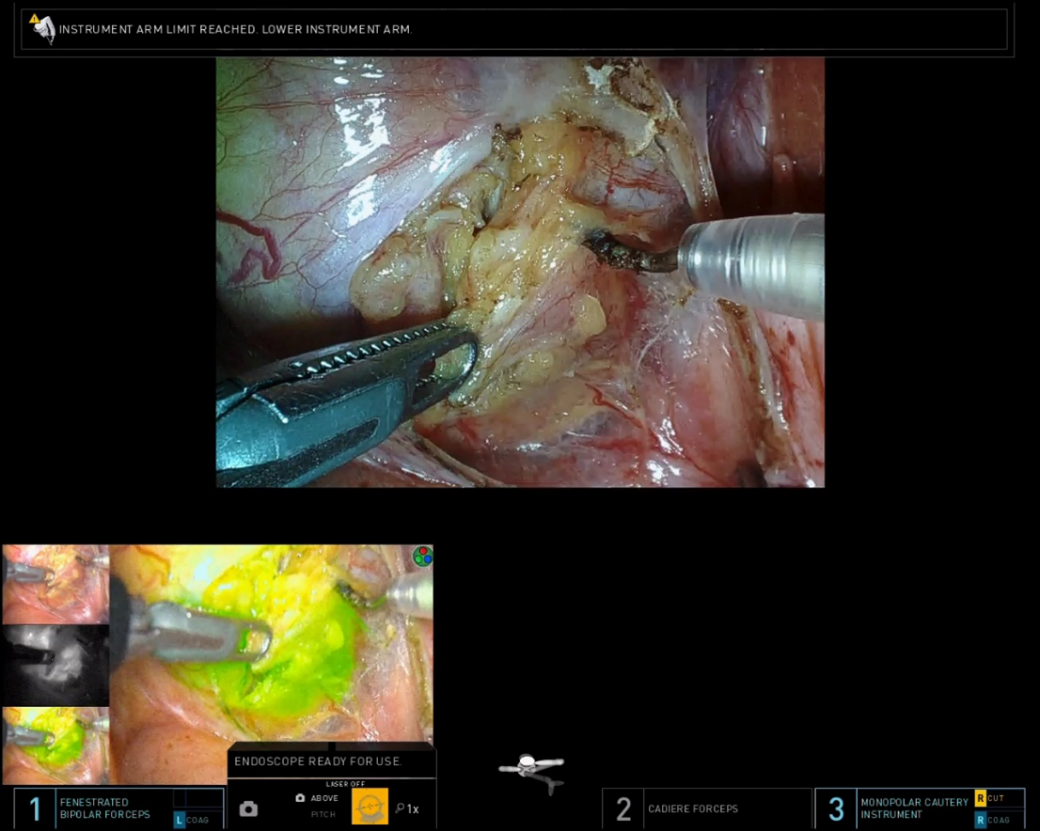


**Supplementary figure 2.**

Assisted imaging system with endoscopic near-infrared fluorescence. The assisting imaging system was connected with SP console monitor during sentinel lymph node biopsy.

*SP*, da Vinci SP system
